# C-reactive protein as an early marker of healthcare-associated pneumonia outcome in cancer patients

**DOI:** 10.1186/2197-425X-3-S1-A299

**Published:** 2015-10-01

**Authors:** L Sarmet Cunha Farah Rabello, T Lisbo, L Azevedo, JR Lapa e Silva, M Soares, P Póvoa, J Salluh

**Affiliations:** Instituto Nacional de Cancer, Rio de Janeiro, Brazil; D'Or Institute for Research and Education, Rio de Janeiro, Brazil; Intensive Care Unit, Santa Casa de Misericórdia de Porto Alegre, Porto Alegre, Brazil; Intensive Care Unit, Hospital Sirio-Libanes, São Paulo, Brazil; Postgraduate Program of Internal Medicine - Universidade Federal do Rio de Janeiro, Rio de Janeiro, Brazil; Unidade Polivalente de Terapia Intensiva, Hospital de São Francisco Xavier, Centro Hospitalar de Lisboa Ocidental, CEDOC, Faculdade Médica NOVA, Nova Universidade de Lisboa, Lisboa, Portugal

## Introduction

Healthcare-associated pneumonia is associated with high mortality rates in critically ill especially those with multidrug-resistant pathogens.

## Objectives

The aim of the present study is to evaluate the value of C-reactive protein (CRP) ratio in the outcome of ICU cancer patients with healthcare-associated pneumonia (HCAP).

## Methods

This was a secondary analysis of a prospective cohort of cancer patients admitted to three ICUs with healthcare-associated pneumonia. CRP was sampled every other day from D0 do D6 of antibiotic prescription. CRP-ratio was calculated in relation to D0 CRP concentration. Comparison between survivors and non-survivors was performed.

## Results

137 patients were included in the study (median age: 65 years; solid tumors 69%; neutropenia 13%). Good performance status was observed in 61% of all patients. The median Charlson comorbidity index was 3 points. Septic shock at ICU admission was present in 74% of patients and invasive mechanical ventilation was used in 73% while 27% used dialysis. 30-day and hospital mortality rates were 54% and 65% respectively.

The course of CRP from D0 to D6 as well as CRP-ratio were significantly different in survivors and non-survivors (p = 0.017 and p = 0.03, respectively). After 48h of antibiotic therapy CRP concentration was significantly lower in survivors (p = 0.042)). By D4, both CPR and CRP-ratio of survivors were significantly lower (p < 0.001 and p = 0.004, respectively). The AUC of CRP and CRP-ratio by D4 in the identification of patients with poor outcome were 0.717 and 0.662, respectively.

## Conclusions

The rate of CRP decrease, assessed either by absolute as well as relative changes, after antibiotic prescription in cancer patients with HCAP was markedly associated with mortality and may be used as an early predictor of outcome.

